# Evaluating the therapeutic potential of different sources of mesenchymal stem cells in acute respiratory distress syndrome

**DOI:** 10.1186/s13287-024-03977-w

**Published:** 2024-10-29

**Authors:** S. Regmi, A. Ganguly, S. Pathak, R. Primavera, S. Chetty, J. Wang, Shaini Patel, A. S. Thakor

**Affiliations:** 1 Interventional Radiology Innovation at Stanford, Department of Radiology, School of Medicine, Stanford University, Stanford, CA 94304 USA; 2 Division of Blood and Marrow Transplantation and Cellular Therapy, School of Medicine, Stanford University, Stanford, CA 94305 USA

**Keywords:** Mesenchymal stem cells, Acute respiratory distress syndrome, Umbilical cord, Inflammation, Immune responses

## Abstract

**Background:**

Mesenchymal stem/stromal cells (MSCs) have attracted interest as a potential therapy given their anti-inflammatory and immunomodulatory properties. However, clinical trials using MSCs for acute respiratory distress syndrome (ARDS) have produced mixed and inconclusive data. In previous work, we performed a “head-to-head” comparison between different sources of MSCs and showed that each source had a unique genomic and proteomic “signature”.

**Method:**

This study investigated which sources of MSC: bone marrow derived-MSCs (BM-MSCs), adipose tissue derived-MSCs (AD-MSCs) and umbilical cord derived-MSCs (UC-MSCs)  would be the optimal candidate to be used as a therapy in an LPS-induced mouse model of ARDS. Immune cells assessment, tissue transcriptomics, animal survival, and endothelial-epithelial barrier assessment were used to evaluate their effects.

**Results:**

When comparing the three most commonly used MSC sources, we found that UC-MSCs exhibited greater efficacy compared to other MSCs in improving animal survival, mitigating epithelial/endothelial damage, decreasing lung inflammation via reducing neutrophil infiltration, T cell proliferation, and M1 polarization. Bulk RNA sequencing of lung tissue also showed that UC-MSCs have the capability to downregulate extracellular trap formation, by the downregulation of key genes like *Elane* and *Padi4*. Notably, treatment with UC-MSCs demonstrated a significant reduction in Fc-γ R mediated phagocytosis, which has been associated with monocyte pyroptosis and intense inflammation in the context of COVID-19.

**Conclusion:**

Our findings suggest that UC-MSCs are an optimal source of MSC to treat acute inflammatory conditions in the lungs, such as ARDS.

**Supplementary Information:**

The online version contains supplementary material available at 10.1186/s13287-024-03977-w.

## Introduction

Despite decades of research, therapies for acute respiratory distress syndrome (ARDS) remain remarkably limited. While there are no approved pharmacological treatments, current strategies include respiratory support with mechanical ventilation, extracorporeal membrane oxygenation (ECMO) to treat refractory hypoxemia, and corticosteroids to treat inflammation [1]. One potential therapy for ARDS is to use mesenchymal stem cells (MSCs) given their anti-inflammatory and immunomodulatory properties [2]. Emerging studies now show MSCs can increase alveolar fluid clearance and promote lung barrier function by restoring the metabolic health of epithelial, endothelial and innate immune cells [2–5]. In different preclinical studies, different sources of MSCs derived from adipose tissue (AD-MSCs) [6], bone marrow (BM-MSCs) [7], placenta [8], and umbilical cord (UC-MSCs) [9] have shown effectiveness in treating ARDS. However, their clinical translation has not been effective as demonstrated by variable outcomes from clinical trials. The results of clinical studies are mixed in terms of disease related markers and overall clinical benefit and patient outcomes. These discrepancies suggest that MSCs derived from different sources could have different therapeutic efficacy in patients with ARDS. This is supported by our recent study demonstrating MSCs from different sources have different regenerative signatures, as reflected by genomic and proteomic analyses [10].

Hence, in the current study we compared the effect of three different sources of MSCs: AD-MSCs, BM-MSCs and UC-MSCs in an LPS-induced mouse model of ARDS. The objective of this study was to characterize the therapeutic effects of MSCs from different sources in the setting of ARDS, with the goal of providing a mechanistic explanation for choosing a specific source of MSC for the treatment of patients suffering from this condition. This study suggests that UC-MSCs possess potent immunomodulatory properties and can effectively reduce inflammation in the respiratory tract. Their mechanism of action enables them to protect and maintain the integrity of epithelial and endothelial cells, thereby preserving the crucial epithelial-endothelial barrier in the lungs. Furthermore, compared to more commonly used BM-MSCs or AD-MSCs, UC-MSCs demonstrate superior efficacy in improving animal survival, indicating their strong potential for clinical translation in the setting of ARDS.

## Materials and methods

### Cell culture—mesenchymal stem cells

Human MSCs from each source (AD-MSCS, BM-MSCs and UC-MSCs) were obtained from StemBioSys (USA) and characterized at passage (P)1, as previously described [10], to ensure they met the criteria determined by the International Society for Cell and gene Therapy (ISCT). In brief, all MSCs were characterized for osteogenesis, adipogenesis, presence of CD73, CD105 and CD90 and absence of CD34, CD45 and MHCII expression as described previously [10]. For each MSC source, a single donor (UC-MSC: Donor 18,001, AD-MSC: Donor 18,002, and BM-MSC: Donor 19,001) at P4-P6, was used for all experiments in the present study. All MSCs were expanded in low glucose DMEM (Hyclone, USA) with 10% fetal bovine serum (FBS) (Hyclone, USA) and 1% penicillin and streptomycin (Hyclone, USA) under 20% O_2_ and 5% CO_2_ at 37 °C.

### ARDS in a murine model

All animal experiments were performed in 8–10 weeks old female C57/BL6 mice (Charles River Laboratories, USA) in accordance with the Administrative Panel on Laboratory Animal Care (APLAC, protocol 33,868) at Stanford University. ARDS was induced via an intra-tracheal (IT) injection of lipopolysaccharide (LPS; Sigma Aldrich, USA) at a dose of 5 mg/kg in phosphate buffered saline (PBS). For IT injection, mice were anesthetized with isoflurane and laryngoscope-guided IT injection was done to ensure the injection into trachea. After 4 h of LPS injection, mice from a cage were randomized to receive a single intravenous injection of 1 × 10^6^ MSCs (from a single source) or PBS. After 48 h, all mice were euthanized using isoflurane followed by cervical dislocation and blood, lungs, and bronchoalveolar lavage (BAL) samples were collected for cytokine, total protein, and immune cell assessment. For the collection of BAL, a catheter or feeding tube was inserted into the trachea. Using a 1 mL syringe, 800 μL of PBS was instilled twice into the bronchial tree and then aspirated. The collected fluid was centrifuged at 500 g for 10 min to settle the cells, and the supernatant was collected for cytokines and protein analysis and cell were used for flow cytometric analysis [11]. This work has been reported in line with the ARRIVE guidelines 2.0.

### Immune cell profiling

BAL was centrifuged at 500 g for 5 min to pellet down cells with the supernatant collected for analysis of cytokines and proteins. From lung tissue samples, single cells were obtained following collagenase (Sigma, USA) digestion at a dose of 1 mg/mL at 37 °C for 1 h. The enzyme activity was stopped with full RPMI and filtered using a 70 µm cell strainer, before being centrifuged at 500 g for 5 min. All single cells obtained were then washed and incubated with FC block for 20 min at 4 °C, before fixative live dead dye (Thermofischer Scientific, USA), fluorescent anti-CD11b, anti-Ly6G, anti-TCR-β, anti-CD86, anti-CD206, anti-CD4, and anti-CD8 antibodies (Biolegend, USA) were added.

### T cell suppression assay

The immune suppressive effect of MSCs was evaluated in T cells isolated from the spleen of C57BL/6 mice. T cells were isolated using a pan T cell isolation kit (Stem Cell Technology) and labeled with cell trace violet (CTV; Thermoscientific, USA) in PBS for 20 min at 37 °C. Next, 1×10^5^ T cells and 1×10^4^ MSCs were co-cultured in a U-shaped 96-well plate for 5 days in the presence of CD3/CD28 activation beads (Miltenyl, USA). Live T cells were then stained with anti-CD3, anti-CD4, and anti-CD8 antibodies for FACS analysis.

### Effect of MSCs on macrophages polarization

Raw 264.7 macrophages were seeded (0.3 × 10^6^/ well) in a 12 well plate and allowed to attach overnight. Next, these cells were treated with 0.5 μg/ml LPS and MSCs (6 × 10^4^/ insert) in transwell inserts at a 1:5 ratio for 36 h. Then, Raw 264.7 cells were collected, washed, centrifuged, and stained for live/dead, anti-CD11b, anti-CD206, and anti-CD86 antibodies for FACS analysis.

### Cellular model of lung injury in A549 cells

A549 cells were cultured with MSCs in a 1:5 ratio (MSCs: A549) in a transwell culture in the presence of inflammatory cytokines: TNF-α (50 ng/ml) and IFN-γ (100 ng/ml). Cell viability was assessed using a CCK-8 reagent according to the manufacturer’s guidelines (GLPBio, USA). For the determination of apoptosis, we used a Cell Death Detection ELISA^PLUS^ (Roche, USA) according to the manufacturer’s guidelines. Absorbance was measured using a microplate reader (Tecan, USA).

### Bulk RNA sequencing, RT-PCR, and secretome analysis

Whole lung tissue was collected and homogenized in trizol (Thermofisher Scientific, USA). RNA extraction was then done using RNA mini kit (Qiagen, Germany) according to the manufacturer’s guidelines with the quality of RNA evaluated using an Agilent bioanlyzer 2100 system. Paired-end 150 bp sequencing was carried out on an Illumina NovaSeq platform. The quality assessment, read filtering and mapping were performed using the NGS QC toolkit and alignment was performed using HISTAT2 tool against Mus Musculus (GRCm39/mm39). HTSeq was used to quantify the reads [12]. For RT-PCR, iTaq Universal One-Step RT-qPCR Kits were used according to the manufacturer’s guidelines. The primers used in this study are listed in Table S1. For secretome analysis, we collected the conditioned media from basal and stimulated MSCs as described previously [10].

### Histological evaluation

Lung tissue for different groups was collected after 48 h and fixed in 4% paraformaldehyde solution. Tissue samples were then embedded in paraffin and sectioned and embedded on glass slides. For hematoxylin and eosin (H&E) staining, the sections were deparaffinized in xylene, rehydrated using an alcohol series, and then stained with hematoxylin and eosin, as described previously [13]. Finally, images were acquired using a NanoZoomer slide scanner 2.0-RS (Hamamatsu, Japan) and FIJI ImageJ software.

### Bioinformatics data processing

DESeq2 (v3.14)[14] were used to perform differential gene expression analysis. Differentially Expressed Genes (DEG) were determined using a significance threshold with a *p*-value < 0.05. Gene enrichment analysis of DEG [15, 16] was performed using The Database for Annotation, Visualization and Integrated Discovery (DAVID) [17, 18]. Heat maps were created utilizing Cluster 3.0 with correlation uncentered data and single linkage clustering.

### Statistical analysis

Statistical analysis was performed using a non-parametric test (Kruskal–Wallis test followed by 2-stage linear step-up procedure of Benjamini, Krieger, and Yekutietli for pairwise comparison or an unpaired *t* test), where indicated, using GraphPad prism 8 software (GraphPad Software, Inc., USA). The number of animal replicates are represented as the individual points/dots unless otherwise specified. All data are expressed as mean ± standard error of mean (SEM) or min to max plot, where a *p* value < 0.05 was considered statistically significant.

## Results

### MSCs improve survival during ARDS by providing protection against epithelial and endothelial cell damage

**Fig. 1:**
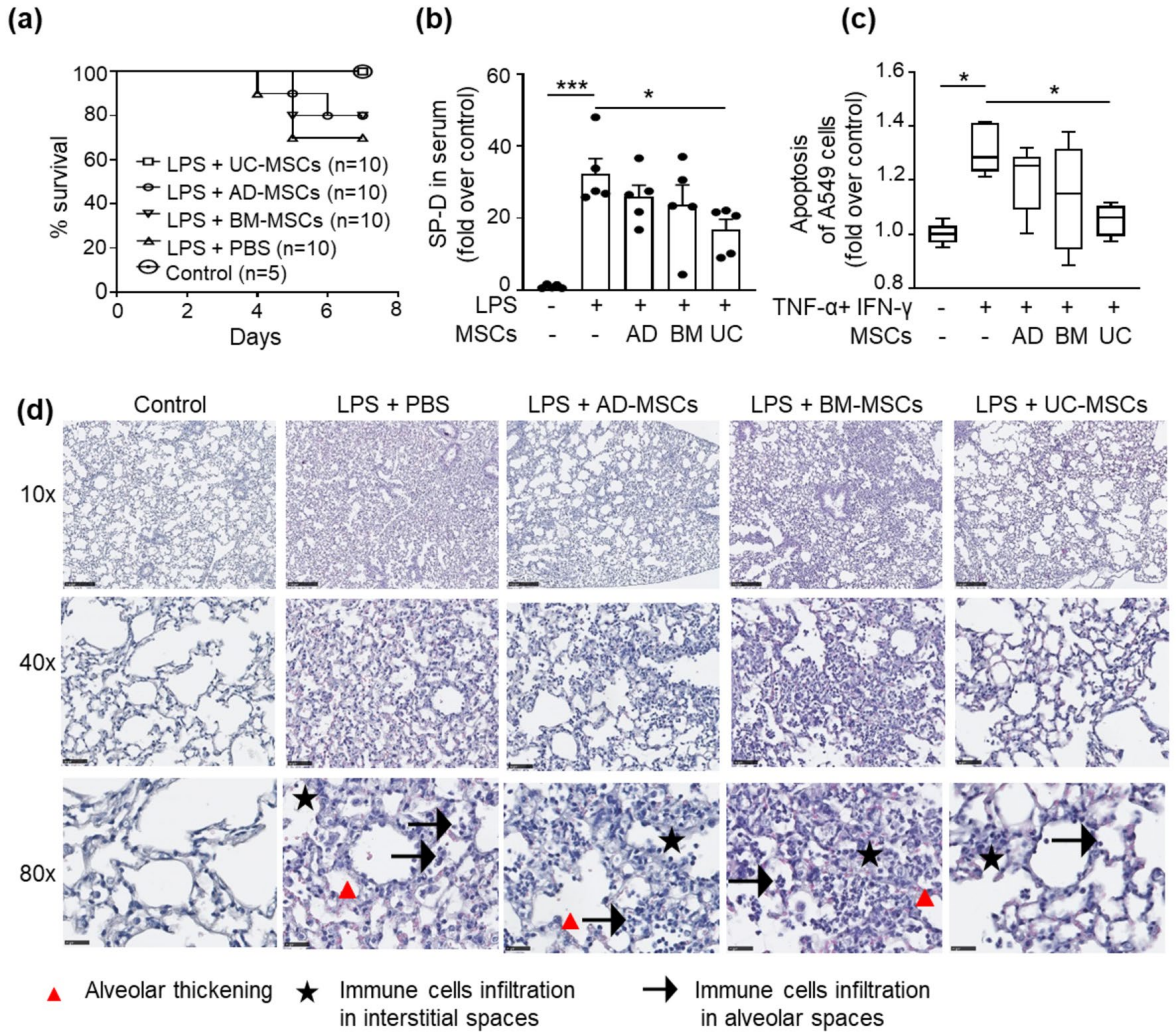
The effect of different sources of MSCs in reducing inflammation and preventing endothelial-epithelial barrier damage. **a** Survival of animals following LPS induced ARDS with and without MSC-therapy. Data is represented as the percentage survival. **b** Levels of serum surfactant protein D (SP-D), 48 h after LPS injection. Data is represented as the mean ± SEM. *p < 0.05, ***p < 0.001; Kruskal–Wallis test, followed by 2-stage linear step-up procedure of Benjamini, Krieger, and Yekutietli for pairwise comparison. Each point represents an independent animal. **c** apoptosis of A549 cells (n = 5) following 48 h exposure to an inflammatory cocktail (TNF-α: 50 ng/ml and IFN-γ: 100 ng/ml). Data is represented as the min to max plot (n = 5). *p < 0.05, **p < 0.01; Kruskal–Wallis test. **d** Representative histology images (H&E staining: red triangle = alveolar thickening; star = inflammatory cells/neutrophil infiltration in the interstitial spaces; arrow = inflammatory cells/neutrophil infiltration in alveolar air spaces). Scale Bar = 250 μm (10x); 50 μm (40x); 25 μm (80x)

ARDS was induced in mice using an IT injection of 5 mg/kg LPS. To validate the induction of the disease, we analyzed key indicators which included serum SP-D, total cell count, total protein content, and IL1β secretion in BAL 4 h post LPS injection. We found a significant increase in serum SP-D, total cells, total protein, and IL1β concentration in BAL, implying successful disease induction. (Figure S1). After 4 h, mice received an intravenous injection of either PBS or MSCs from different sources. LPS induced significant inflammation in the lungs as demonstrated on CT imaging which showed bilateral hazy infiltrates [19], predominately in the dorsal aspect of the lungs (Figure S2a), with a corresponding reduction in animal survival over 7 days (Fig. 1a). Following LPS, there was also an increase in serum SP-D level (Fig. 1b), due to damage of the alveolar-capillary interface. The increased permeability was also validated with Evans blue dye which showed higher dye retention in the lungs following LPS (Figure S2b-c) and increased total protein in BAL (Figure S2d). Following treatment with different sources of MSCs, CT imaging appearances of the lung were only partially improved with AD-MSCs and BM-MSCs, but markedly improved with UC-MSCs, which also showed improvement in survival with levels comparable to control (non-LPS treated animals) (Fig. 1a).Treatment with MSCs also reduced total protein in BAL and SP-D in serum in the following order: UC-MSC > BM-MSC > AD-MSC (Fig. 1b and S2d). To further validate the protective effect of MSCs on lungs epithelial cells during inflammation, proof-of-concept in vitro experiments were also performed with human alveolar epithelial cell line (A459 cells) in inflammatory conditions using TNF-α, and IFN-γ, which are important pro-inflammatory cytokines that drive LPS-induced lung damage. The results show a significant reduction in apoptosis of epithelial cells when these cells were cultured with UC-MSCs following exposure to inflammatory stimuli (Fig. 1c and S2e). Furthermore, the weight of the lungs was also slightly reduced in animals treated with UC-MSCs (Figure S2f), again suggesting their ability to reduce parenchymal inflammation and edema, especially since UC-MSCs reside in the lung following intravenous administration (Figure S2g). This was supported by histological evaluation of lung tissue which showed that following LPS treatment there was marked alveolar wall thickening and neutrophil infiltration into the alveolar and interstitial spaces that was reduced with MSC therapy, with the effects most prominent in the UC-MSC group (Fig. 1d).

## MSCs reprogram the innate immune system to attenuate lung inflammation

To assess the therapeutic potential of MSCs in reducing inflammation in ARDS affected lungs, we evaluated cytokines in lung tissue samples following LPS administration. Here, we observed an increase in mRNA expression of *TNF-α* and *IL-6*. Following treatment with MSCs, these pro-inflammatory cytokines were significantly reduced with UC-MSCs (Figure S3a and b). On the other hand, although no difference was noted in *IL-10* mRNA expression following LPS, animals treated with UC-MSCs showed a moderate increase in this gene expression, though this difference was not statistically significant (Figure S3c).

**Fig. 2:**
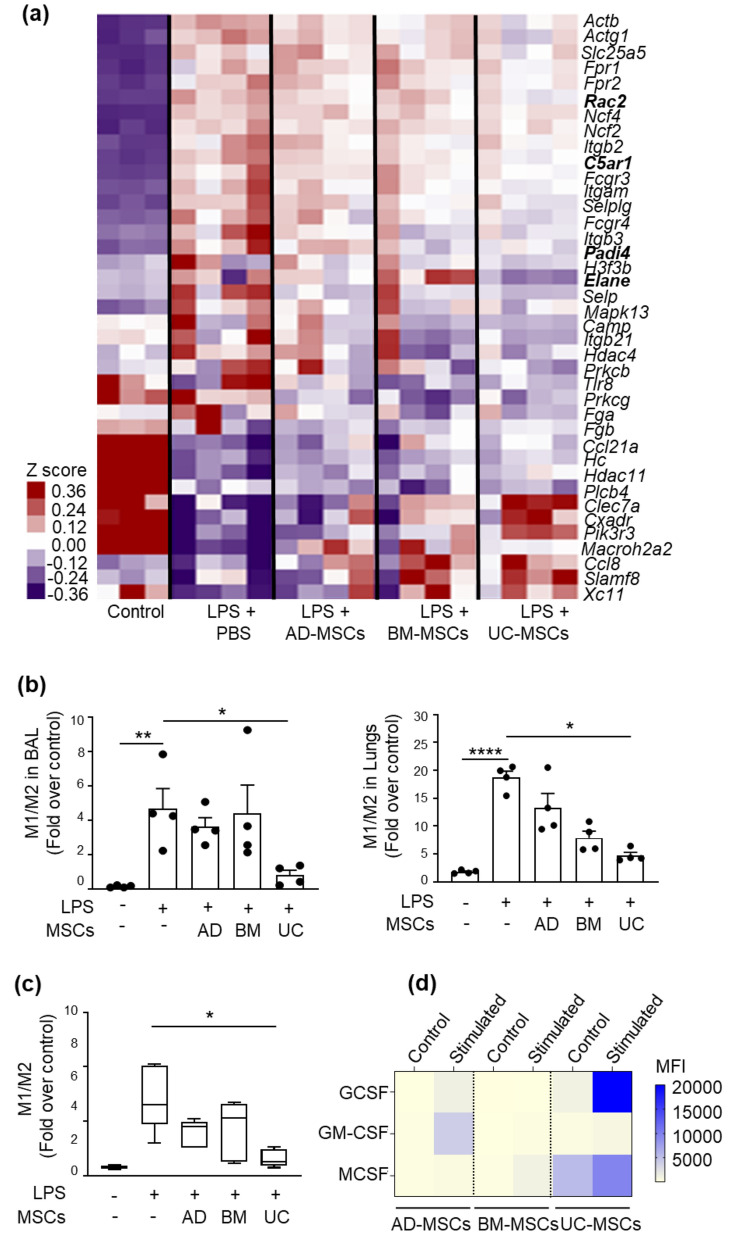
The effect of different sources of MSCs in reprograming the innate immune system. **a** Heatmap showing differential expression of genes related to neutrophil extracellular trap (NET) formation following LPS-induced ARDS with/without MSC-therapy. **b** Changes in M1/M2 (CD11b^+^CD86^+^/CD11b^+^CD206^+^) ratio in BAL and lungs tissue. Data is represented as the mean ± SEM. *p < 0.05, **p < 0.01; Kruskal–Wallis test, followed by 2-stage linear step-up procedure of Benjamini, Krieger, and Yekutietli for pairwise comparison. Each point represents an independent animal. **c** In vitro assessment of M1/M2 ratio in Raw 264.7 cells with/without different sources of MSCs before and after stimulation with LPS. Data is represented as the min to max plot (n = 5). *p < 0.05; Kruskal–Wallis test, followed by 2-stage linear step-up procedure of Benjamini, Krieger, and Yekutietli for pairwise comparison. **d** GCSF, GM-CSF and MCSF secretion in the conditioned media from three different sources of MSCs in their basal and stimulated conditions (i.e. following treatment with an inflammatory cocktail (TNF-α: 50 ng/ml and IFN-γ: 100 ng/ml) for 48 h). Data is represented as the mean of two independent replicates from same donor.

Next, we assessed the changes in infiltration of neutrophils and monocyte/macrophages in the lung; these are the major first line immune cells activated during inflammation. Our transcriptomic data of lung tissue also showed downregulation of neutrophil extracellular trap formation (NET) pathway with downregulation of genes, such as *Rac2*, *Elane*, *Padi4,* and *C5ar1* in animals treated with UC-MSCs, which was not evident in animals treated with AD-MSCs and BM-MSCs (Fig. 2a, Data S1). Our data indicates a significant increase in neutrophil infiltration into the lung tissue following LPS, which was slightly reduced following MSC treatment this effect being slightly better with BM-MSCs and UC-MSCs  (Figure S4a). Myeloperoxidase (MPO) activity, which represents activated neutrophil activity, was also reduced with MSC treatment (Figure S4b) but this effect could not reach statistical significance. No significant effect was seen in neutrophil recruitment in the BAL following any MSC treatment (Figure S4c). In addition to the effect on neutrophils, LPS-induced ARDS also caused a significant infiltration of monocytes (Figure S4d and S4e), accompanied by an increase in their pro-inflammatory M1 phenotype relative to an anti-inflammatory M2 phenotype (Fig. 2b), in both the BAL and lung tissue. While MSC treatment did not affect monocyte recruitment, it significantly promoted their M2 phenotype, with this effect being more predominant with BM-MSCs and UC-MSCs. Similar results were observed with our proof-of-concept in vitro model, where murine-monocyte cells (Raw264.7) showed a statistically significant decrease in the M1/M2 ratio, following co-culture with UC-MSCs (Fig. 2c). Furthermore, UC-MSCs had a higher expression of anti-inflammatory factors (i.e. MCSF and GCSF) associated with polarization of monocytes to an anti-inflammatory M2 phenotype [10] (Fig. 2d).

## MSCs inhibit T cell infiltration and proliferation

**Fig. 3:**
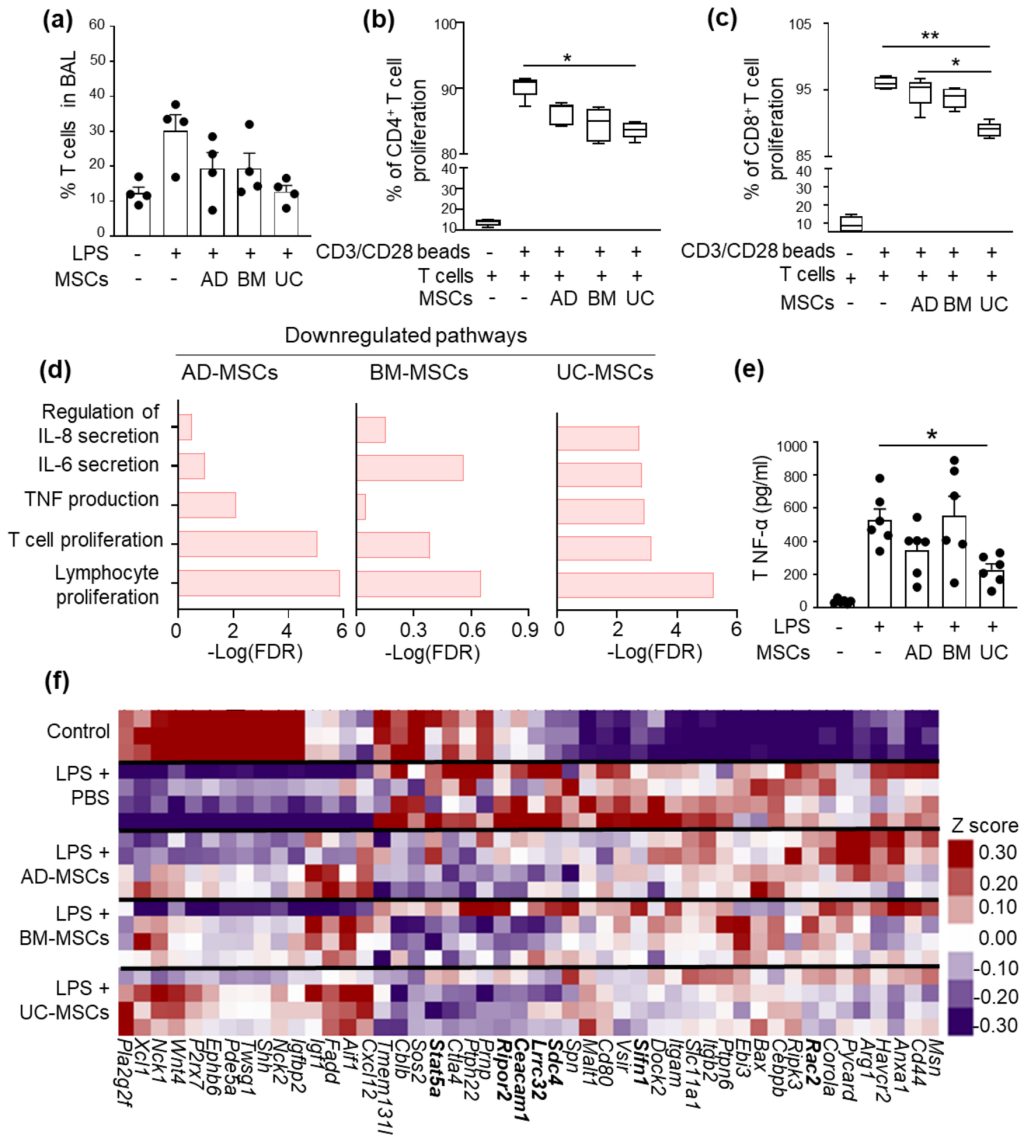
The effect of different sources of MSCs on T-cell infiltration and proliferation. **a** Percentage of total T cell infiltration (TCR-β + cells) in the BAL. Data is represented as the mean ± SEM. Kruskal–Wallis test. Each point represents an independent animal. In vitro assessment of **b** CD4^+^ and **c** CD8^+^ T cell proliferation with/without different sources of MSCs. Data is represented min to max plot (n = 4). *p < 0.05, **p < 0.01; Kruskal–Wallis test, followed by 2-stage linear step-up procedure of Benjamini, Krieger, and Yekutietli for pairwise comparison. Gene ontology (GO) enrichment analysis showing pathways related to T cell proliferation that are downregulated in **d** AD-MSCs, BM-MSCs and UC-MSCs compared to PBS. **e** TNFα secreted in BAL. Data is represented as the mean ± SEM. *p < 0.05; Kruskal–Wallis test, followed by 2-stage linear step-up procedure of Benjamini, Krieger, and Yekutietli for pairwise comparison. **f** Heatmap showing differential expression of genes related to T-cell proliferation following LPS-induced ARDS with/without MSC-therapy

While the innate immune system plays a vital role in initiating inflammation, it is the adaptive immune response which is responsible for the continued and sustained damage to lung tissue. Hence, to explore the effect of MSCs on the adaptive immune system, we evaluated T lymphocytes in both the lung tissue and BAL. In LPS-induced ARDS animals, there was no significant increase in the total T cell population (TCR-β^+^ cells) within lung tissue (Figure S4f), but there was an increase in the total T cells in the BAL, which was reduced with UC-MSC treatment, however, this effect could not reach statistical significance (Fig. 3a). Similarly, while LPS caused a reduction in CD4^+^/CD8^+^ ratio in both the BAL (Figure S4g) and lungs (Figure S4h), recovery was only noted in the lungs following UC-MSC therapy (Figure S4h). In in vitro studies, a T cell proliferation assay showed that all three sources of MSCs exhibited suppressive effects on the proliferation of both CD4^+^ and CD8^+^ T cells, with UC-MSCs showing a greater suppressive effect (Fig. 3b-c). Interestingly, the pathways for the regulation of IL-8 secretion, IL-6 secretion, TNF-alpha production, T cell proliferation and lymphocyte proliferation were significantly downregulated following UC-MSC treatment, compared to other sources of MSCs (Fig. 3d, Data S2). This was further validated with ELISA where there was significant reduction in TNF-α production in the BAL with UC-MSCs treatment compared to other MSCs and PBS group (Fig. 3e). Moreover, genes involved in lymphocyte proliferation pathway, viz *Rac2*, *Sifn1*, *Sdc4*, *Lrrc32*, *Stat5a*, *Ceacam1*, and *Ripor2,* also showed downregulation in their expression following UC-MSC treatment, compared to AD-MSCs and BM-MSCs (Fig. 3f). Transcriptomic analysis of lung tissue showed down regulation of all of these pathways, and when taken together, this may be responsible for the T cell suppressive effect observed in vitro and in vivo with UC-MSCs, compared to other sources of MSCs.

### UC-MSCs downregulate pathways responsible for development of ARDS

**Fig. 4:**
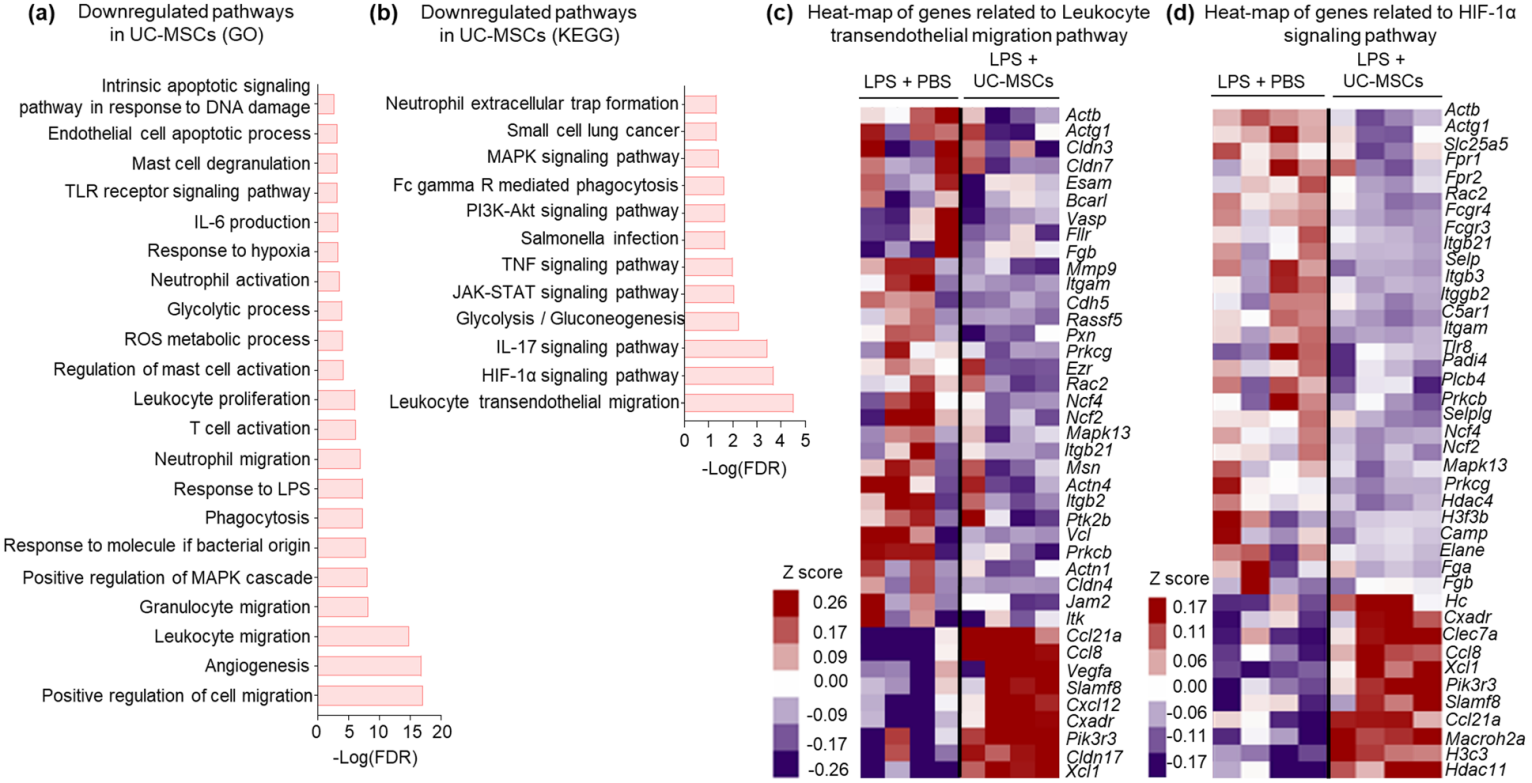
Transcriptomic enrichment analysis of lung samples showing the effect of UC-MSC-therapy. **a** Gene Ontology (GO) and **b** Kyoto Encyclopedia of Genes and Genomes (KEGG) enrichment of significantly (FDR < 0.05) DOWN regulated pathways in UC-MSCs treatment group compared to LPS. Heatmap showing differential gene expression for pathways related to **c** leukocyte trans-endothelial migration and **d** HIF-1α signaling comparing LPS-induced ARDS with/without UC-MSC therapy

We performed bulk RNA sequencing of lung tissue and investigated different pathways that are UP/DOWN regulated during ARDS and compared their significance following MSC therapies. Both GO and KEGG pathway analysis was performed which indicated an upregulation of different inflammatory pathways such as mast cell degranulation, leukocyte proliferation, granulocyte/neutrophil and leukocyte migration, toll like receptor (TLR) signaling, phagocytosis, TNF and IL-17 signaling, NF-kappa B signaling, and NK cell mediated cytotoxicity in LPS-induced ARDS animals compared to healthy controls. Moreover, in LPS-induced ARDS animals the pathways for intrinsic and extrinsic apoptotic signaling, via ROS production and HIF-1α signaling, were also significantly upregulated (Figure S5a-b, Data S1-3). Following treatment with UC-MSCs, these inflammatory and apoptotic pathways were downregulated both in GO (Fig. 4a, Data S2) and KEGG (Fig. 4b, Data S4) enrichment analyses compared to LPS-induced ARDS animals. AD-MSC and BM-MSC treated groups also showed downregulation of these pathways, however, they were more effectively downregulated in animals treated with UC-MSCs (Fig. 4a-b, S5c-d, Data S2-3). Furthermore, the differential expression of genes related to leucocyte migration (Fig. 4c) and HIF-1α (Fig. 4d) following UC-MSC therapy also signified their role in mitigating inflammation and hypoxia, respectively, thereby addressing the adverse effects of LPS-induced ARDS.

## Discussion

In the current study, we evaluated the efficacy of three different sources of MSCs (AD-MSCs, BM-MSCs, and UC-MSCs) in mitigating the adverse pathophysiological effects encountered in ARDS. We found that UC-MSCs have better efficacy with significantly improved therapeutic effects when compared to other sources of MSCs. Indeed, UC-MSCs showed a significant reduction in respiratory tract inflammation while concurrently protecting epithelial and endothelial cells, thereby allowing them to maintain a functional barrier within the lungs. Gene expression profiling provided further mechanistic insights into how UC-MSCs are better suited to attenuate lung inflammation and modulate immune responses. These data have implications for potentially choosing UC-MSCs as the main source of MSC for further clinical translation and testing in the setting of ARDS.

In the present study, we have mainly focused on the early exudative phase of ARDS. The exudative phase of ARDS starts with accumulation of neutrophils in the lungs contributing to the increased production of ROS that interrupts the alveolar-capillary barrier and promotes the permeability of protein-rich fluid into the alveolar lumen, which subsequently results in pulmonary edema [20]. Activated neutrophils produce neutrophil extracellular traps (NETs), which are net like structures composed of DNA-histone complexes and proteases such as elastase (NE) and myeloperoxidase (MPO), that have a crucial role in pathogen clearance [21]. However, prolonged or inappropriate activation of neutrophils will produce too much NET that then contributes to the pathogenesis of ARDS with an aggravated inflammatory response [22], characterized by NE damaging vascular endothelial cells and compromising their integrity [23], and MPO generating reactive oxygen species (ROS) that locally mediates alveolar damage [24]. Indeed, dysregulated NETs result in severe inflammation that correlates with disease severity [25, 26] that promotes the formation of Nucleotide-binding domain Leucine-rich-containing Repeat and Pyrin domains (NLRPs) and the further production of inflammatory cytokines such as IL-1β and IL-8 [27]. In our study, we observed a significant reduction in the infiltration of neutrophils when using BM-MSC and UC-MSC therapies. However, only UC-MSCs showed a reduction in NE expression in lungs, and overall NET formation compared to the PBS-treated control group. Our transcriptomic analysis also showed that UC-MSCs caused a significant reduction in the gene expression of *Elane* (which is responsible for NE production) and *Padi4* (which contributes to NET formation); the regulation of these genes and proteins by UC-MSCs reduce the overall formation of NET and hence pulmonary tissue damage, resulting in improved survival and lower inflammatory responses.

In addition, there is monocyte infiltration with polarization to an M1 (i.e. inflammatory) phenotype and a corresponding decrease in M2 polarization (i.e. anti-inflammatory phenotype) [28]. The over-activated monocytes also activate NLRP3, which then triggers pyroptosis that further aggravates ARDS [29]. Several studies have shown that if monocytes can be shifted in polarization from an M1 (a pro-inflammatory) to an M2 (an anti-inflammatory) phenotype, this has the ability to suppress cytokine storms, resolve inflammation, promote tissue repair, and prevent ARDS-related mortality [30, 31]. Our data suggests that UC-MSCs can promote polarization of monocytes to an M2 phenotype more effectively when compared to other sources of MSCs resulting in the attenuation and resolution of lung inflammation. Moreover, our study demonstrated that UC-MSCs, unlike other MSCs, can significantly reduce Fc-γ R mediated phagocytosis which is particularly significant considering the recent COVID-19 pandemic where Fc-γ R mediated uptake of the SARS-COV-2 virus was responsible for monocyte pyroptosis and the subsequent development of severe inflammation [32].

In ARDS, the breakdown of the epithelial-endothelial leads to an increased permeability that causes pulmonary edema and ultimately respiratory failure. In our study, we observed that treatment with UC-MSCs reduced permeability into the alveolar space. This effect is likely due to the protective impact of UC-MSCs on lung epithelial cells, achieved by decreasing ROS production and enhancing cell survival during inflammatory conditions. Furthermore, the downregulation of NET formation, suppression of CD4^+^ and CD8^+^ T cell mediated toxicity, and inhibition of M1 polarization of monocytes in the UC-MSC-treated animals collectively inhibit with the pathological processes that drive ARDS. As a result, UC-MSC treatment helps to preserve the integrity of the alveolar epithelial and vascular endothelial barriers, thus mitigating the progression of ARDS and improving outcomes. Hence, in the present work, we believe that UC-MSCs attenuate ARDS through their immunomodulatory and regenerative effects. However, they could also work through other avenues that were not specifically evaluated in the present work given that other studies have shown that UC-MSCs also have the ability to differentiate into type 2 alveolar epithelial cells (AEC2) [33], as well as play an important role in regulating the differentiation of type II alveolar epithelial cells (ACEII) by inactivating Yes-associated protein (YAP) [34].

Despite promising preclinical outcomes, several clinical trials have used MSCs in patients with ARDS, from different sources, and have not shown a significant improvement. Indeed, the START prospective, double-blind, multicenter, randomized phase 2a study, in which one dose of BM-MSCs was given intravenously, was shown to be safe in patients with moderate to severe ARDS, but demonstrated no difference in 28-day mortality [35]. Recently, a large phase 2 clinical trial conducted in moderate to severe ARDS from COVID-19, using two infusions of BM-MSCs, also showed no improvement in 30-day survival or 60-day ventilator-free intervention [36]. In another double-blind randomized trial in COVID-19 related ARDS, two intravenous infusions of UC-MSCs resulted in an improvement in patient survival and recovery time with an associated reduction in inflammation [37, 38]. Another phase 1 clinical trial also suggested that UC-MSCs significantly reduced inflammation without any severe side effects following a single IV injection in COVID-19 related ARDS patients [39–41]. Moreover, long-term assessment of UC-MSC therapy in COVID-19 patients demonstrated their safety and effectiveness in treating severe COVID-19 infection [42]. However, another multicenter, double-blind, randomized trial in COVID-19 associated mild-to-severe ARDS suggested that there was no significant difference in P_a_O_2_/FiO_2_ after an intravenous injection of UC-MSCs [43] from day 0 to day 7. The variability in results may be attributed to the diverse sources of MSCs, each possessing unique properties and effects. These differences likely influence their therapeutic potentials, underscoring the challenges in MSC standardization. Beyond the established standards that define MSCs, there remains a critical need for further standardization in the procedures for MSC isolation, expansion, characterization, and application. This would help minimize discrepancies in MSC-based therapies, and boost the reproducibility and effectiveness of therapeutic outcomes across various studies and clinical interventions. Moreover, different dosing strategies and use of MSCs in different etiologies and severities of ARDS make drawing definitive conclusions somewhat difficult. Additionally, the scarcity of preclinical data comparing various MSC sources in the treatment of ARDS further emphasizes the need for comparative studies in different preclinical models. Nevertheless, our data shows that UC-MSCs were mechanistically superior to other sources of MSCs for the treatment of LPS-induced ARDS in mice with findings that closely mirror those reported using this source of MSC in clinical trials.

In our in vitro and in vivo experiments, we observed that UC-MSCs were able to better promote M2 macrophage polarization and T cell suppression compared to other sources of MSCs. In addition, UC-MSCs demonstrated an enhanced ability to maintain epithelial-endothelial barrier function by preventing apoptosis in these cells. While these findings underline the superior effect of UC-MSCs in treating ARDS, they have implications for other diseases where UC-MSCs maybe more effective in immune regulation and/or maintaining epithelial-endothelial barrier, such as acute kidney injury, inflammatory bowel disease, systemic lupus erythematosus, multiple sclerosis, and diabetes.

One limitation of the current study is that the animals were treated with MSCs during the early phase of the disease. Future studies will examine the therapeutic effect of UC-MSCs administered in later phases of disease progression. Multiple MSC administrations might also be considered to augment their effects. While the present work focused on understanding the mechanism of action in mitigating the acute insult, future studies will focus on the longer-term effects of using UC-MSCs to mitigate the chronic effects of ARDS which can include lung fibrosis with reduced lung function. Although we have shown there is limited donor variability between UC-MSCs [10], future studies will also test additional donors, as well as different gender animals from different age groups and different ARDS animal models.

## Conclusion

In conclusion, comprehensive evaluation of the efficacy of the most commonly used MSCs (i.e. AD-MSCs, BM-MSCs, and UC-MSCs) to treat ARDS reveals superiority of UC-MSCs in mitigating LPS-induced ARDS in a murine model. UC-MSCs exhibited enhanced immunomodulatory effects, particularly in promoting macrophage polarization towards an anti-inflammatory phenotype, as well as in suppressing NET formation and T cell proliferation. Our findings advocate for the preferential utilization of UC-MSCs as an optimal MSC source for combating acute inflammatory conditions, such as ARDS.

## Supplementary Information

Additional file 1.

Additional file 2.

Additional file 3.

Additional file 4.

Additional file 5.

Additional file 6.

## Data Availability

The authors declare that all data supporting the findings of this study are available within the article and its supplementary material files. The transcriptomics data has been deposited in GEO with the data set identifier GSE241186.

## Abbreviations

AD-MSCs: Adipose tissue derived-MSCs
ARDS: Acute respiratory distress syndrome
BAL: Bronchoalveolar lavage
BM-MSCs: Bone marrow derived-MSCs
COVID-19: Coronavirus disease 2019
CT: Computed tomography scan
ECMO: Extracorporeal membrane oxygenation
ELISA: Enzyme-linked immunosorbent assay
EVs: Extracellular vesicles
Fc-γ R: γ Subunit of the immunoglobulin Fc receptor
GCSF: Granulocyte colony stimulating factor
GO: Gene Ontology
HIF-1α: Hypoxia-inducible factor 1-alpha
IFN-γ: Interferon gama
IL-10: Interleukin 10
IL-1β: Interleukin-1 beta
IL-6: Interleukin 6
IL-8: Interleukin 8
IV: Intravenous
KEGG: Kyoto encyclopedia of genes and genomes
LPS: Lipopolysaccharide
MCSF: Macrophage colony-stimulating factor
MPO: Myeloperoxidase
MSCs: Mesenchymal stem cells
NE: Neutrophil elastase
NETs: Neutrophil extracellular traps
NLRP3: Nucleotide-binding domain, leucine-rich–containing family, pyrin domain–containing-3
PBS: Phosphate buffered saline
ROS: Reactive oxygen species
RT-PCR: Reverse transcription polymerase chain reaction
SARS-COV-2: Severe-acute-respiratory-syndrome-related coronavirus-2
SP-D: Surfactant protein D
TLR: Toll like receptor
TNF-α: Tumor necrosis factor alpha
UC-MSCs: Umbilical cord derived-MSCs

## Declarations

### Resources availability

Corresponding Author: Further information and requests for resources should be directed to and will be fulfilled by the corresponding author, Dr. Avnesh S Thakor (asthakor@stanford.edu).
