# Supplementary material for: Evaluating the therapeutic potential of different sources of mesenchymal stem cells in acute respiratory distress syndrome
Source: Stem Cell Res Ther. 2024 Oct 29;15:385. doi: 10.1186/s13287-024-03977-w (PMC11520775; doi:10.1186/s13287-024-03977-w)
Supplement: Supplementary file 4 — Additional file 4. [file 13287_2024_3977_MOESM4_ESM.pdf]

Evaluating the therapeutic potential of different sources of mesenchymal stem cells in acute respiratory distress syndrome

S. Regmi[1], A. Ganguly[1], S. Pathak[2], R. Primavera[1], S. Chetty[1], J. Wang[1], Shaini Patel[1], and A. S. Thakor[1*]

[1]Interventional Radiology Innovation at Stanford, Department of Radiology, Stanford University, School of Medicine, Stanford, CA 94304, USA

[2]Division of Blood and Marrow Transplantation, Stanford University, School of Medicine, Stanford, CA 94305, USA

[*]*A. S. Thakor is the corresponding author of this work. e-mail: asthakor@stanford.edu*

Supplementary Methods

Western blot

For western blot analysis, MSCs were cultured with A549 cells in a 1:5 ratio (MSCs:A549 cells) for 48 h in the presence of inflammatory cytokines TNF-α (50 ng/ml), and IFN-γ (100 ng/ml). The cells were collected, washed twice with Dulbecco's phosphate-buffered saline (DPBS) and centrifuged at 12,000 g at 4°C for 15 min to obtain the cell pellet. The cells were lysed using RIPA buffer with a protease inhibitor and quantified using Pierce BCA Protein assay kit (Thermo-scientific). Next, 20 µg of protein was separated using SDS-PAGE, and transferred to a nitrocellulose membrane using a Trans-Blot Turbo System (Bio-rad). The membrane was blocked using 5% BSA in TBST (Tris-Buffered Saline with 0.1% Tween 20) for 1 h in room temperature and then incubated overnight at 4 °C with a primary antibody for caspase 3 antibody (Cell signaling) and GAPDH or Actin (Cell Signaling) in 3% BSA in a dilution of 1:1000. The membrane was then washed with TBST, incubated with secondary antibody for 1 h at room temperature, and finally washed to observe the chemoluminescence of the respective protein.

Pulmonary microvascular permeability

Evans blue dye extravasation technique was used to evaluate pulmonary microvascular permeability as described previously [2]. Briefly, after 48 h of LPS injection, Evans blue (20 mg/kg; Sigma-Aldrich) was injected intravenously via the tail vein. After 30 min, lungs were perfused with PBS containing 5 mM ethylenediaminetetraacetic acid through the right ventricle to remove any intravascular dye. Next, the lungs were kept at 60 °C for 36 h with formamide (Sigma-Aldrich) for extravasation assessment of the Evans Blue dye based on the weight of tissue. The formamide solution was centrifuged and the supernatant evaluated for dye detection at 610 nm wavelength using a microplate reader (Tecan, USA).

Computerized Tomography (CT)

For cross sectional imaging of the lungs, mice were anesthetized and scanned using a micro (µ)-CT data-acquisition system (VivaCT 40, Scanco) at 38 µm voxel size (70 kV, 57µA, 273 ms integration time). Image reconstruction was performed using the Inveon Research workplace 4.2 (Siemens Medical Solutions, USA).

Biodistribution of MSCs

UC-MSCs were stained with VivoTrack680 (PerinElmer, USA) according to manufacturer's protocol. One million UC-MSCs were injected intravenously and the ex-vivo imaging of different organs were done after 24 h of cell injection using Lago optical imaging system.

Supplementary Figures

FigureS1

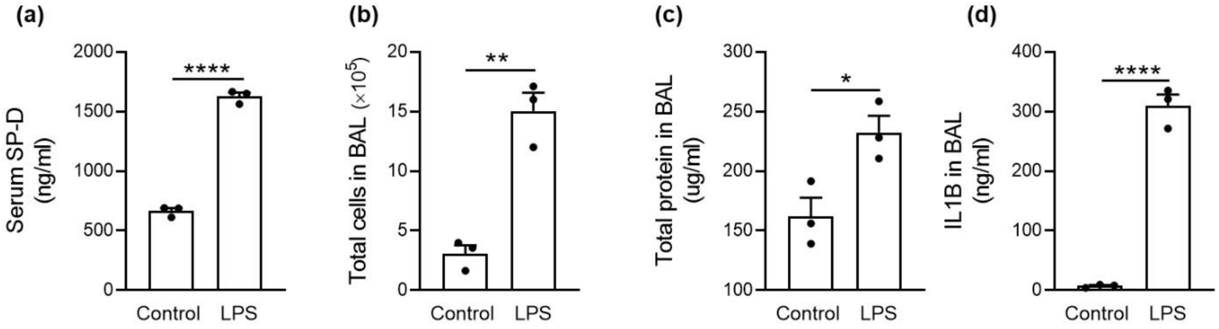

Figure S1. Induction of ARDS using intra-tracheal injection of LPS. After 4h following LPS injection, serum and BAL were collected from animals. In serum, **(a)** SP-D concentration was measured. In BAL, **(b)** total cells, **(c)** total protein, and **(d)** IL1B concentration were measured. Each point represents an individual animal. The data represents mean ± SEM (n=3). *p < 0.05, ** p<0.01, ****< 0.0001 in unpaired T test.

FigureS2

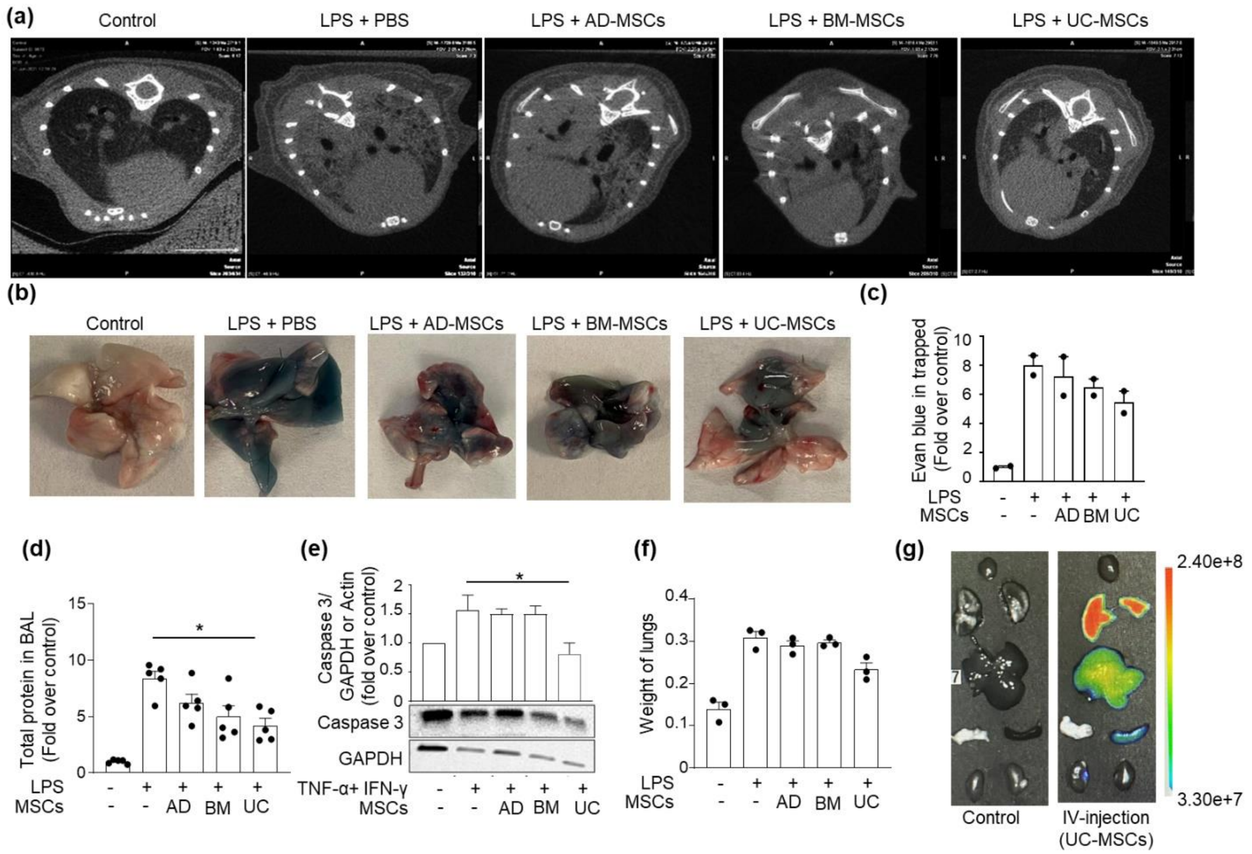

Figure S2. The protective effect of MSCs in reducing endothelial-epithelial barrier damage **(a)** CT images showing axial section of lung showing inflammation at 48 h after IT injection of LPS. **(b)** Representative images of lung tissue from mice after Evans Blue tail vein injection in the five different groups showing Evan blue entrapped in the lungs with **(c)** their respective quantification. Each point represents the individual animal. **(d)** Total protein in BAL. The data represents mean ± SEM (n=3). *p < 0.05 in Kruskal-Wallis test, followed by 2-stage linear step-up procedure of Benjamini, Krieger, and Yekutietli for pairwise comparison. **(e)** Caspase 3 expression using Western blot analysis after *in vitro* culture of MSCs with A549 cells in transwell for 48 h in inflammatory condition. The data represents mean ± SEM (n=3). *p < 0.05 in Kruskal-Wallis test, followed by 2-stage linear step-up procedure of Benjamini, Krieger, and Yekutietli for pairwise comparison. An example western blot is also shown. **(f)** The weight of isolated lungs from different groups. The data represents ± SEM, each point represents the individual animal). **(g)** Ex-vivo biodistribution of VivoTrack680 labelled UC-MSCs 24 h after an IV injection via the tail vein measured using Lago optical imaging system.

Figure S3

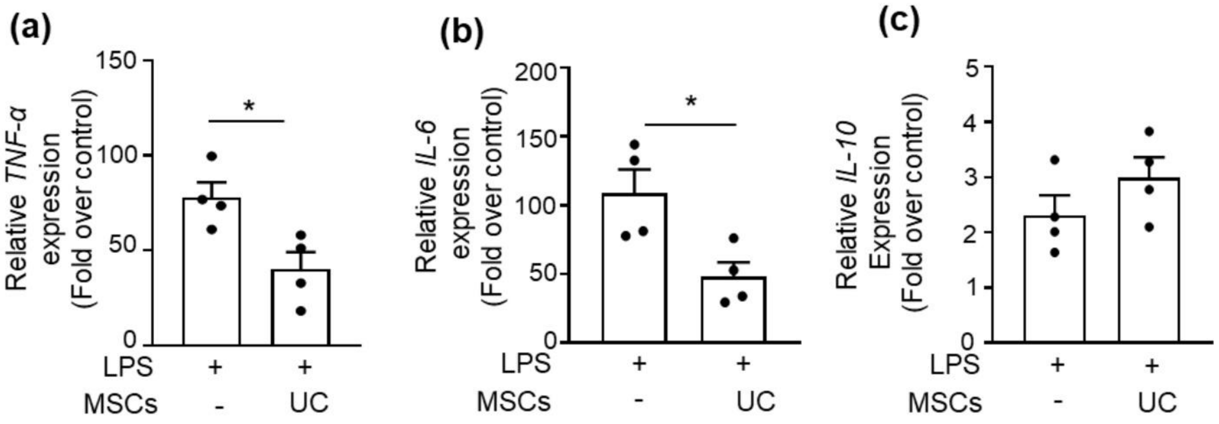

Figure S3. The effect of UC-MSCs in inhibiting the expression of inflammatory cytokines in the lung. Relative mRNA expression of **(a)** *TNF-α*, **(b)** *IL-6*, and (**c**) *IL-10* in the lungs 48 h after LPS injection. GAPDH was used as the housekeeping gene to normalize the mRNA expression of the genes. Data is represented as the mean ± SEM. *p<0.05; non-parametric unpaired t-test. Each point represents an independent animal.

Figure S4

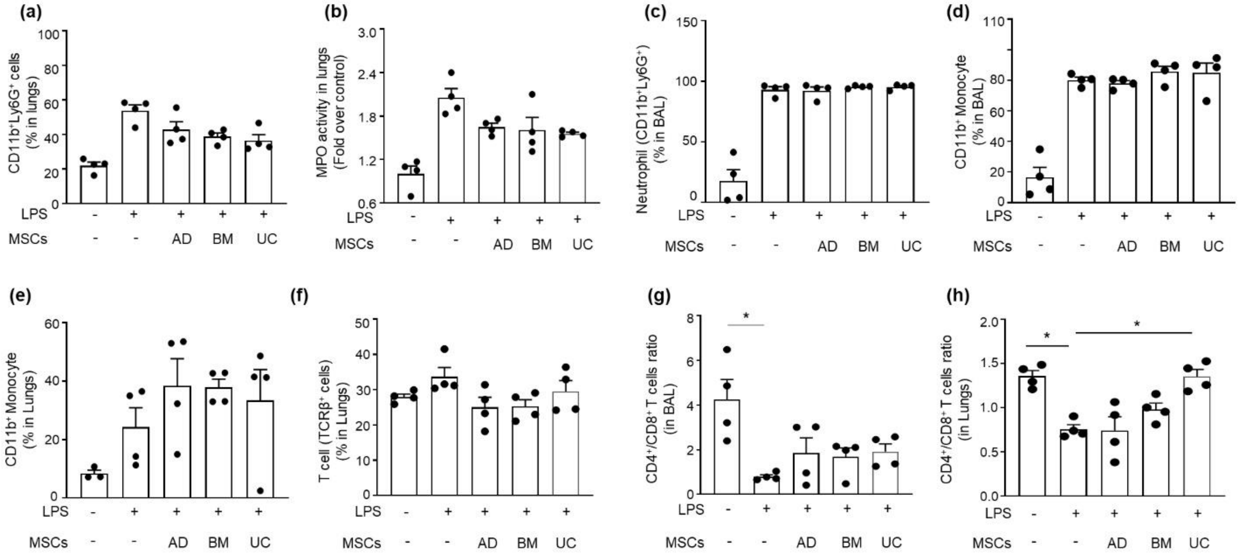

Figure S4. **The effect of MSCs on immune cells during lung inflammation. (a)** Percentage of neutrophils ($CD11^+Ly6G^+$ cells) and **(b)** MPO activity in lungs tissue. Percentage of **(c)** neutrophil and **(d)** monocyte (CD11b+ cells), and **(e)** $CD4^+/CD8^+$ T cells ratio in BAL. Percentage of (**f**) total monocyte **(g)** total T cells in lungs and **(h)** $CD4^+/CD8^+$ T cells in lungs. Data represents mean ± SEM (*$p < 0.05$, in Kruskal-Wallis test, followed by 2-stage linear step-up procedure of Benjamini, Krieger, and Yekutietli for pairwise comparison, each point represents the individual animal).

Figure S5

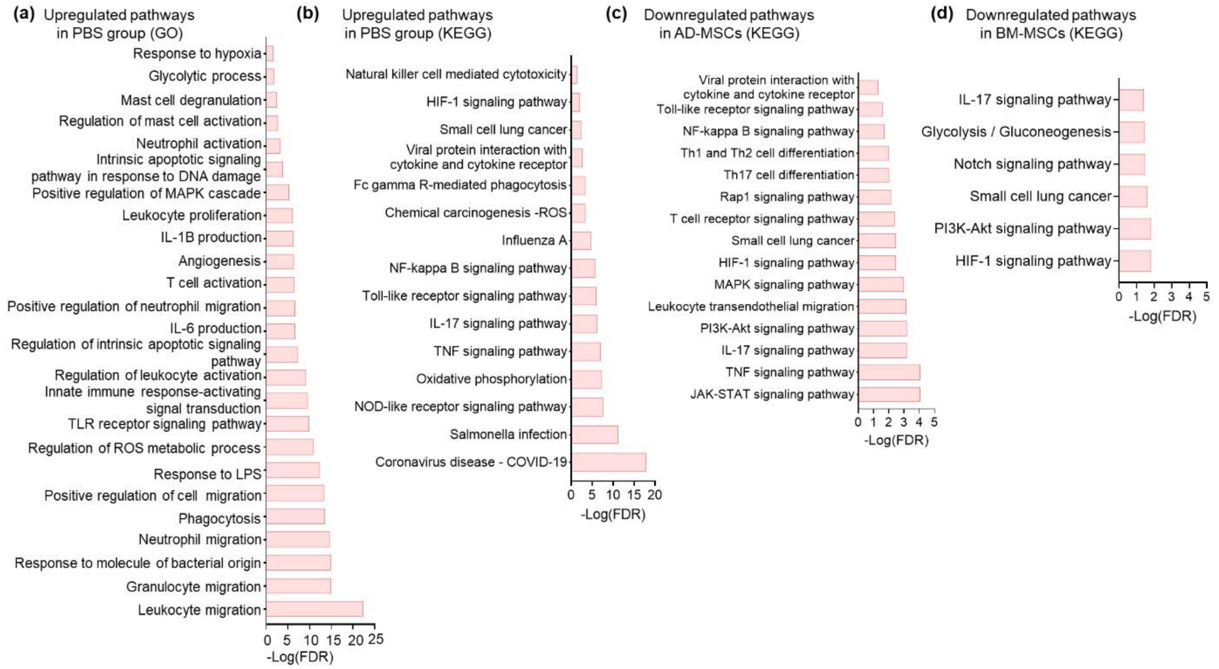

Figure S5. **Transcriptomic enrichment analysis. (a)** GO enrichment and **(b)** KEGG enrichment indicating significant (FDR < 0.05) pathways upregulated in LPS compared to control. (**c-d**) KEGG enrichment indicating significant (FDR < 0.05) pathway downregulated in **(c)** AD-MSCs and **(d)** BM-MSCs compared to PBS.

Table S1: Primer sequence used in qPCR

**Primer Name**	**Sequence (5'-3')**
TNF-F1	TAGCCCACGTCGTAGCAAACC
TNF-R1	GGTGAGGAGCACGTAGTCGG
IL 6-F1	TACCACTTCACAAGTCGGAGGC
IL 6-R1	CTGCAAGTGCATCATCGTTGTTC
IL 10-F1	AGTGATGCCCCAGGCAGAGA
IL 10-R1	GACACCTTGGTCTTGGAGCTTAT
GAPDH-F1	CATCACTGCCACCCAGAAGACTG
GAPDH-R1	ATGCCAGTGAGCTTCCCGTTCAG

Data S1: mRNA transcriptomic- FPKM and DEG values

Data S2: GO pathway analysis

Data S3: KEGG pathway analysis

1. Ganguly A, Swaminathan G, Garcia-Marques F, et al. Integrated transcriptome-proteome analyses of human stem cells reveal source-dependent differences in their regenerative signature. *Stem Cell Reports* 2023;18(1):190-204.
2. Reutershan J, Morris MA, Burcin TL, et al. Critical role of endothelial CXCR2 in LPS-induced neutrophil migration into the lung. *J Clin Invest* 2006;116(3):695-702. doi: 10.1172/jci27009 [published Online First: 2006/02/18]
