# Supplementary material for: Evaluating the therapeutic potential of different sources of mesenchymal stem cells in acute respiratory distress syndrome
Source: Stem Cell Res Ther. 2024 Oct 29;15:385. doi: 10.1186/s13287-024-03977-w (PMC11520775; doi:10.1186/s13287-024-03977-w)
Supplement: Supplementary file 5 — Additional file 5. [file 13287_2024_3977_MOESM5_ESM.docx]

**Evaluating the therapeutic potential of different sources of mesenchymal stem cells in acute respiratory distress syndrome**

S. Regmi^1^, A. Ganguly^1^, S. Pathak^2^, R. Primavera^1^, S. Chetty^1^, J. Wang^1^, Shaini Patel^1^, and A. S. Thakor^1*^

^1^Interventional Radiology Innovation at Stanford, Department of Radiology, Stanford University,

School of Medicine, Stanford, CA 94304, USA

^2^Division of Blood and Marrow Transplantation, Stanford University, School of Medicine, Stanford, CA 94305, USA

*^*^A. S. Thakor is the corresponding author of this work. e-mail:* [*asthakor@stanford.edu*](mailto:asthakor@stanford.edu)

| Primer Name | **Sequence (5'-3')** |
| --- | --- |
| TNF-F1 | TAGCCCACGTCGTAGCAAACC |
| TNF-R1 | GGTGAGGAGCACGTAGTCGG |
| IL 6-F1 | TACCACTTCACAAGTCGGAGGC |
| IL 6-R1 | CTGCAAGTGCATCATCGTTGTTC |
| IL 10-F1 | AGTGATGCCCCAGGCAGAGA |
| IL 10-R1 | GACACCTTGGTCTTGGAGCTTAT |
| GAPDH-F1 | CATCACTGCCACCCAGAAGACTG |
| GAPDH-R1 | ATGCCAGTGAGCTTCCCGTTCAG |

**Table S1. Primer sequence**
