# Supplementary material for: Evaluating the therapeutic potential of different sources of mesenchymal stem cells in acute respiratory distress syndrome
Source: Stem Cell Res Ther. 2024 Oct 29;15:385. doi: 10.1186/s13287-024-03977-w (PMC11520775; doi:10.1186/s13287-024-03977-w)

Evaluating the therapeutic potential of different sources of mesenchymal stem cells in acute respiratory distress syndrome

S. Regmi[1], A. Ganguly[1], S. Pathak[2], R. Primavera[1], S. Chetty[1], J. Wang[1], Shaini Patel[1], and A. S. Thakor[1*]

[1]Interventional Radiology Innovation at Stanford, Department of Radiology, Stanford University, School of Medicine, Stanford, CA 94304, USA

[2]Division of Blood and Marrow Transplantation, Stanford University, School of Medicine, Stanford, CA 94305, USA

A. S. Thakor is the corresponding author of this work. e-mail: asthakor@stanford.edu

Full western blots for figure S1e

Caspase 3 (SET-1)

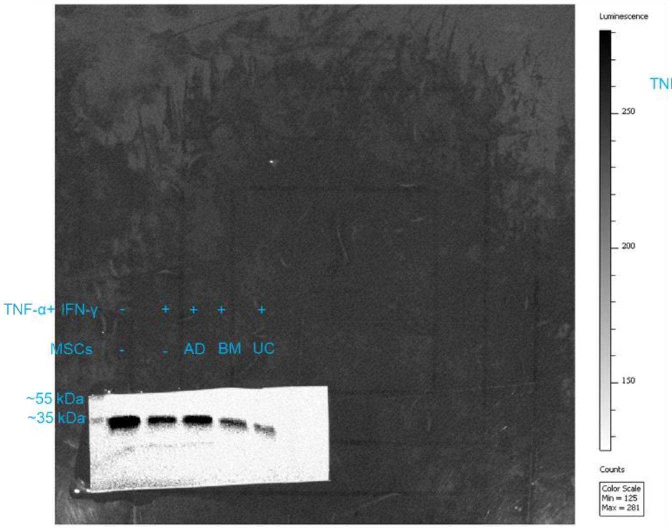

Housekeeping protein: GAPDH (SET-1)

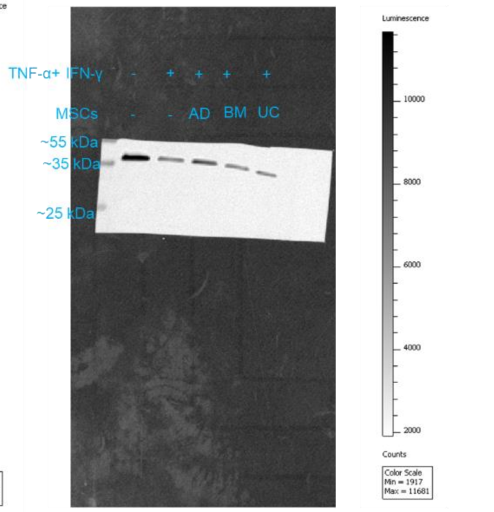

Caspase 3 (SET 2 and 3)

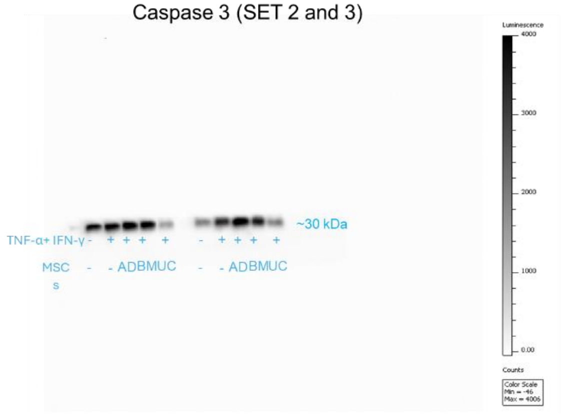

Housekeeping protein: Actin (SET2 and 3)

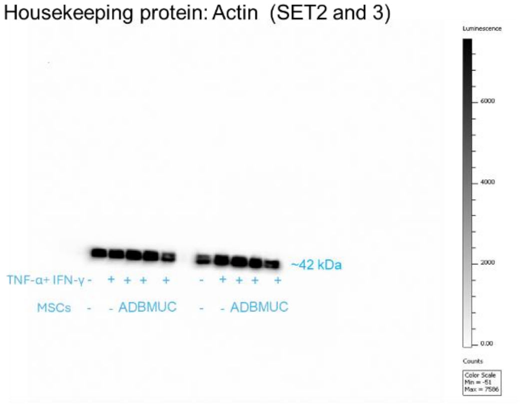

Supplement: Supplementary file 6 — Additional file 6. [file 13287_2024_3977_MOESM6_ESM.pdf]
